# Dental Electro-Therapeutics

**Published:** 1914-03-15

**Authors:** 


DENTAL ELECTRO-THERAPEUTICS. Under this title Lea and Febiger have just issued a valuable manual, which is Edited by Ernest Sturridge, L. D. S., D. D. S., of England. It is a work of 300 pages. The first part of the book is devoted to a statement of the science of Electro-physics as applied to dentistry from the Electroscope to the X-ray machine. The second and perhaps the most important part of the book details the Electro-therapeutic used in dentistry of this agent and physiologic statement and the technical application is most interestingly made. The theory of Ionic Medication is clearly stated and the subject is made very interesting because of the successful application of this form of Medication to peridental tissues. If it could be definitely controlled there is no question, but it could be used to great advantage in all forms of peridentities. The Authors methods of treating pyorrhea by electrolysis has been advocated among the numerous other treatments for this disease, but so far with only varying success.

There is more hope for good results in the various dental treatments from the high frequency current in stimulating trophic changes and this chapter of the book is worthy of careful consideration. It is a valuable edition to our literature and every worker in Electro-physics should possess it.

